# Progress on structural modification of Tetrandrine with wide range of pharmacological activities

**DOI:** 10.3389/fphar.2022.978600

**Published:** 2022-08-16

**Authors:** Liuying Mo, Fan Zhang, Feng Chen, Lei Xia, Yi Huang, Yuemi Mo, Lingqiu Zhang, Daquan Huang, Shunli He, Jiagang Deng, Erwei Hao, Zhengcai Du

**Affiliations:** ^1^ Guangxi Scientific Experimental Center of Traditional Chinese Medicine, Guangxi University of Chinese Medicine, Nanning, China; ^2^ Guangxi Collaborative Innovation Center of Study on Functional Ingredients of Agricultural Residues, Nanning, China; ^3^ Guangxi Key Laboratory of Efficacy Study on Chinese Materia Medica, Nanning, China; ^4^ Guangxi International Zhuang Medicine Hospital Affiliated to Guangxi University of Chinese Medicine, Nanning, China; ^5^ Office of the President, Guangxi University of Chinese Medicine, Nanning, China; ^6^ Guangxi Dahai Sunshine Pharmaceutical, Nanning, China; ^7^ Guangxi Heli Pharmaceutical, Nanning, China

**Keywords:** tetrandrine (TET), poor water solubility, low bioavailability, structure modification, nanocarrier delivery systems

## Abstract

Tetrandrine (Tet), derived from the traditional Chinese herb Fangji, is a class of natural alkaloids with the structure of bisbenzylisoquinoline, which has a wide range of physiological activities and significant pharmacfological effects. However, studies and clinical applications have revealed a series of drawbacks such as its poor water solubility, low bioavailability, and the fact that it can be toxic to humans. The results of many researchers have confirmed that chemical structural modifications and nanocarrier delivery can address the limited application of Tet and improve its efficacy. In this paper, we summarize the anti-tumor efficacy and mechanism of action, anti-inflammatory efficacy and mechanism of action, and clinical applications of Tet, and describe the progress of Tet based on chemical structure modification and nanocarrier delivery, aiming to explore more diverse structures to improve the pharmacological activity of Tet and provide ideas to meet clinical needs.

## 1 Introduction

Natural products play a key role in drug discovery, and many of the widely used contemporary drugs are derived from natural products. For example, paclitaxel, found in the bark of the gymnosperm redbud, and pergolide, found in the periwinkle plant of the oleaceae family, are widely used as clinical chemotherapy drugs for the treatment of pancreatic cancer, non-small cell lung cancer, breast cancer, acute leukemia, and many other cancers (Khan et al., 2022), Penicillin, found in Penicillium, is a commonly used antibiotic in clinical practice. Chinese medicine is the accumulation of thousands of years of practical experience of the Chinese people in the application of natural products for the treatment of diseases, and is a treasure trove of natural product discovery. For example, artemisinin, which has significant efficacy against malaria, and berberine, which has significant efficacy against tumors and diabetes, etc., have been discovered from Chinese medicine. The continuous development of new natural products, especially those from traditional Chinese medicine, is of great significance to modern medicine. However, natural products often suffer from poor solubility and low bioavailability, so the discovery of natural products with therapeutic effects and modification to make them more suitable for clinical use are important aspects of drug development to enable their application.

The root of *Stephania Tetrandra* S. Moore, known as Fangji in China, is a traditional Chinese medicine, has long been used in the treatment of rheumatism and paralysis, edema and foot pain, urinary discomfort, eczema, and sores, and other conditions. Research on its chemical composition shows that it contains alkaloids, flavonoids, volatile oils, sterols, organic acids and other components. It is rich in alkaloids, mainly containing dozens of alkaloids such as Tetrandrine, Fangchinoline, Stephenanthrine, etc. Among them, Tetrandrine is one of the most concerned active ingredients, and is also considered to be one of the main ingredients for its medicinal effects. Tetrandrine (Tet), also known as powdered alkaloids, is a bisbenzylisoquinoline alkaloid. Tet has a variety of pharmacological activities, including anti-tumor, anti-inflammatory, anti-cellular fibrosis, cardiovascular protection as well as antioxidant, antiviral, and immune enhancement. It protects the cardiovascular system through hypotension, antiarrhythmia, and anti-myocardial ischemia and reperfusion injury (Yu et al., 2001; Pinelli et al., 2010; Xu et al., 2010; Huang et al., 2011; Zhang et al., 2017a; Li et al., 2020), in addition to its antibacterial (Lee et al., 2012; Li et al., 2021) and activation of mesenchymal stem cells to enhance immune regulation (Yang et al., 2016).

Currently, clinical administration of Tet is mainly by oral administration and injection, but there are several problems in its application, such as poor water solubility, low oral bioavailability, short half-life (Que et al., 2019), and poor pharmacokinetic profile (Tian et al., 2016a), the need for high doses leading to poor patient compliance (6–15 tablets per day) (Zhao et al., 2013), gastrointestinal and renal damage (Liang et al., 2010), and toxic effects on liver and lung effects (Tainlin et al., 1982; Yan et al., 2006; Jin et al., 2011; Shi et al., 2016a; Tian et al., 2016b; Chu et al., 2022), among others. In addition, Tet has poor solubility in physiological environments due to the presence of quaternary ammonium salts (Tet saturation = 0.015 mg/ml in pH 7.4 phosphate buffered saline) (Liu et al., 2018). Structural modification of Tet to improve its aqueous solubility and bioavailability, reduce side effects and enhance its toxic effect on cancer cells is an urgent issue to be addressed. In this paper, a comprehensive and systematic discussion of the structural modification of Tet is presented to provide ideas for exploring more diverse structures to improve the pharmacological activity of Tet as well as to meet clinical needs.

## 2 Pharmacological research and clinical application of Tetrandrine

### 2.1 Anti-tumor efficacy of Tetrandrine

**FIGURE 1:**
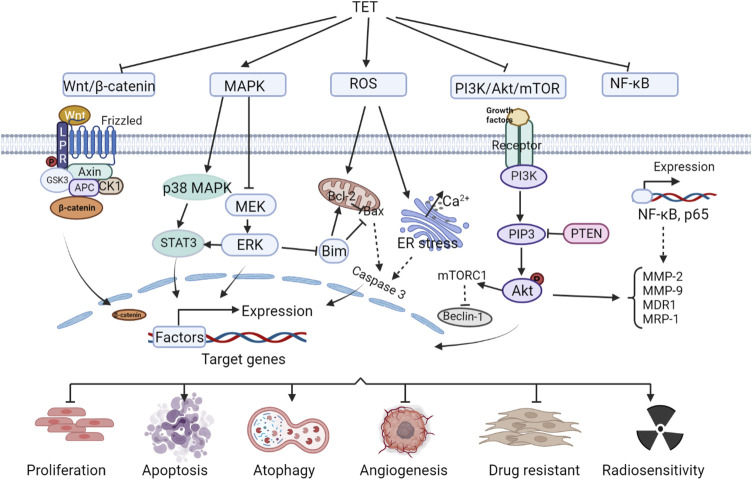
Anti-tumor molecular mechanism of Tet.

Tet has good antitumor effects against a variety of solid tumors and leukemias, including: nasopharyngeal carcinoma (Wang et al., 2020a),bladder cancer (Li et al., 2011a),triple-negative breast cancer (Yuan et al., 2018),lung cancer (Chen et al., 2018),liver cancer (Chen et al., 2018; Zhang et al., 2018),gastric cancer (Wei et al., 2007)and leukemia (Hu et al., 2020), etc. It inhibits tumor cell migration and invasion, enhances radiosensitivity, induces apoptosis, inhibits tumor cell proliferation and tumor growth, induces autophagy, inhibits vascular regeneration, and reverses tumor multidrug resistance. The anti-tumor efficacy and anti-tumor molecular mechanisms of Tet are comprehensively summarized in Figure 1 and Table 1.

**TABLE 1:** Tet anti-tumor types and mechanism of action.

	Cancer type	*In vivo*/*in vitro*	Effect	Mechanism of action	References
1	Nasopharyngeal carcinoma	*in vitro*	Inhibition of migration and invasion of nasopharyngeal carcinoma cell line NPC-TW-039		Wu et al. (2020)
		*in vivo*+*in vitro*	It significantly enhanced the growth inhibition of nasopharyngeal carcinoma cell lines CNE1, CNE2 and C666-1 *in vitro*, enhanced the radiosensitivity of transplanted tumor *in vivo*, and increased the apoptosis rate of transplanted tumor cells induced by radiation	Inhibiting MEK/ERK pathway and inducing autophagy	Wang et al. (2020a)
		*in vitro*	Enhancement of radiosensitivity of nasopharyngeal carcinoma cells CNE1 and CNE2	Elimination of radiation-induced G2/M phase arrest by activating CDC25C/CDK1/Cyclin B1 pathway	Wang et al. (2018)
		*in vitro*	Induction of apoptosis in human nasopharyngeal carcinoma cell line NPC-TW-076	Apoptosis is induced by reactive oxygen and endoplasmic reticulum stress signal pathway	Lin et al. (2016)
		*in vitro*	Induction of apoptosis in human nasopharyngeal carcinoma NPC-TW-039 cells	Apoptosis is induced by calcium-mediated endoplasmic reticulum stress and caspase pathway	Liu et al. (2017a)
2	Bladder cancer	*in vitro*	Inhibition of growth and induction of apoptosis of human bladder cancer cells 5,637 and T24	Caspase cascade and activation of mitochondrial pathway	Li et al. (2011a)
		*in vitro*	Induction of apoptosis in human bladder cancer 5,637 cells and T24 cells	Regulation of AMPK/mTOR signal transduction pathway induces autophagy in human bladder cancer cells, which is helpful to induce apoptosis	Kou et al. (2017)
		*in vitro*	Blocking the migration and invasion of bladder cancer 5,637 cells and T24 cells and reversing the epithelial-mesenchymal transition of bladder cancer	Down-regulation Gli-1	Zhang et al. (2016a)
3	Breast Cancer	*in vivo*+*in vitro*	Inhibition of cell line MDA-MB-231 cell proliferation and reduction of tumor volume and weight	S phase arrest, autophagy and necrotic cell death	Bo et al. (2018)
		*in vitro*	Inhibit the proliferation of inflammatory breast cancer cell line SUM-149 and non-inflammatory metaplastic breast cancer cell line SUM-159, and inhibit the formation of breast glomeruli		Xu et al. (1925)
		*in vitro*	Inhibition of MDA-MB-231 cell proliferation and induction of autophagy	Inhibition of PI3K/AKT/mTOR pathway	Guo and Pei, (2019)
		*in vivo*+*in vitro*	Inhibition of angiogenesis and metastasis in breast cancer	p-ERK↑、NF-κB↓, regulating metastasis and angiogenesis related proteins	Gao et al. (2013b)
		*in vitro*	Reduce the cytoplasm, damage the cell membrane and induce apoptosis of MDA-MB-231 cells	Caspase activation pathway mediated by reactive oxygen species	Gao et al. (2019)
		*in vivo*+*in vitro*	Inhibition of tumor growth and induction of MDA-MB-231 cell apoptosis	Up-regulation of Caspase-3, Bax and Bid, down-regulation of Bcl-2, Survivin and PARP	Wang et al. (2020b)
		*in vitro*	Enhancement of sensitivity of breast cancer cell line MDA-MB-231 to arsenite	Induction of S-phase arrest, apoptosis/necrosis and autophagy death	Yu et al. (2020)
		*in vitro*	Restore the sensitivity of tamoxifen-resistant breast cancer cell line TAM-R to tamoxifen	Inhibition of autophagy and enhancement of apoptosis-promoting effect of tamoxifen	Wang et al. (2021a)
4	lung cancer	*in vitro*	Increased sensitivity of human lung adenocarcinoma PC14 cells to gefitinib	Inhibition of lysosomes	(Sinya)
		*in vitro*	Cytotoxicity to human lung cancer A549 cells	Inhibition of ATP production from mitochondria	Chow et al. (2019)
		*in vitro*	Inhibition of proliferation and induction of apoptosis of A549 human lung cancer cells	Blocking Akt activation selectively inhibits the proliferation of lung cancer cells, inhibits ERK and promotes apoptosis	Cho et al. (2009)
		*in vitro*	Induction of apoptosis and growth arrest in human lung cancer cells	Associated with induction of the Cdk inhibitor p21, inhibition of cell cycle protein D1 and activation of cystatin-3	Lee et al. (2002)
		*in vitro*	Inhibits the growth of lung cancer and induces apoptosis	VEGF/HIF-1α/ICAM-1 signaling pathway	Zhuo et al. (2018)
5	Liver Cancer	*in vitro*	Inhibition of metastasis of human liver cancer cells	Inhibits Wnt/β-catenin pathway activity and reduces metastatic tumor antigen 1 (MTA1) expression	Zhang et al. (2018)
		*in vitro*	Enhanced radiosensitization of human hepatocellular carcinoma cell lines HepG2 and LM3	Attenuation of apoptosis and cell cycle G-phase arrest mediated at least in part by PA28γ	Zhao et al. (2018)
		*in vitro*	Inhibition of hepatocellular carcinoma cell proliferation	Targeting Ca/calmodulin-dependent protein kinase II (camkii δ)	Tha et al. (2019)
		*in vivo*+*in vitro*	Combination therapy with sorafenib showed good synergistic antitumor effects	Reactive oxygen species (ROS)/Akt signaling mediated	Wan et al. (2013)
		*in vitro*	Inhibiting the growth of hepatocellular carcinoma cells Huh-7	Inhibits G2/M phase cell cycle progression and increases caspase-3 expression in cells	Ho, (2013)
		*in vivo*+*in vitro*	Induction of intracellular reactive oxygen species (ROS) accumulation and autophagy	ERK signaling pathway	Gong et al. (2012)
		*in vitro*	Induces apoptosis in hepatocellular carcinoma cells with altered cell morphology, chromatin breakage and caspase activation	Activate reactive oxygen species and inhibit Akt activity	Liu et al. (2011)
		*in vitro*	Combination of nedaplatin significantly enhances apoptosis induction	Regulates the cell cycle, enhances apoptosis induction, and is regulated by multiple genes	Deng et al. (2008)
		*in vivo*+*in vitro*	Increase the sensitivity of hepatocellular carcinoma cells to sorafenib	Inactivate PI3K/AKT/mTOR	Niu et al. (2022)
6	Colon Cancer	*in vivo*+*in vitro*	Induction of apoptosis and inhibition of tumor growth in colon cancer cells	at least partially associated with activation of the p38MAPK signaling pathway	Wu et al. (2010)
		*in vitro*	Inhibits the adhesion, migration and invasion of human colon cancer SW620 cells	Inhibition of nuclear factor-κB, matrix metalloproteinase-2 and matrix metalloproteinase-9 signaling pathways	Juan et al. (2018)
		*in vivo*+*in vitro*	Induction of SW620 apoptosis and inhibition of tumor growth in colon cancer cells	Upregulation of BMP9, and thus inactivation of PI3K/Akt at least by upregulation of PTEN	Zhou et al. (2021)
		*in vitro*	Inhibit the proliferation of colon cancer cells	Bcl-2/Caspase 3/PARP pathway and G1/S phase	Li et al. (2019)
		*in vitro*	Inhibition of proliferation and induction of apoptosis in HCT116 cells	upregulation of TGF-β1 to inactivate PI3K/Akt signaling to reduce PTEN phosphorylation to mediate	Chen et al. (2017a)
		*in vivo*+*in vitro*	Inhibits LoVo cell proliferation and induces apoptosis to inhibit tumor growth	mediated by down-regulation of IGFBP-5 expression, thereby inactivating Wnt/β-catenin signaling	Wu et al. (2015)
		*in vivo*+*in vitro*	Anti-angiogenic effect on LoVo cell transplanted tumors in nude mice	Inhibits cell proliferation, migration and tubular formation, induces apoptosis and inhibits DNA synthesis	Qian et al. (2008)
		*in vivo*+*in vitro*	Enhances the killing effect of radiation on tumor cells both *in vivo* and *in vitro*	Blocking radiation-induced G2 phase block	Sun et al. (2008)
		*in vitro*	Inhibition of the proliferation of human colon cancer cells HCT116	Inhibition of cells in G (1) by convergent mechanisms, including downregulation of E2F1 and upregulation of p53/p21(Cip1)	Meng et al. (2004)
7	Prostate Cancer	*in vitro*	Inhibition of apoptosis of PC3 and DU145 in prostate cancer cells	ROS-mediated, both internal and external pathways	Chaudhary and Vishwanatha, (2014)
		*in vitro*	Inhibition of cell migration and invasion of prostate cancer DU145 and PC3 cells	Negative regulation of Akt/mTOR/MMP-9 signaling pathway	Yang and Zheng, (2016)
		*in vitro*	Improved sensitivity of prostate cancer cells to TRAIL-induced apoptosis	Up-regulation of mRNA expression and protein levels of death receptors Apo Trail R1 (DR4) and Apo Trail R2 (DR5)	Shishodia et al. (2018)
		*in vitro*	Inhibits the proliferation of human prostate cancer cells DU145 and PC-3, induces apoptosis, and inhibits their migration and invasion	Activates caspase cascade and inhibits phosphatidylinositol 3-kinase-Akt signaling pathway to induce apoptosis in a dose-dependent manner	Liu et al. (2015b)
8	Ovarian Cancer	*in vitro*	Significantly enhances cisplatin-induced cell growth inhibition and apoptosis, and causes redistribution of the cell cycle	Regulation of the Wnt/cadherin signaling pathway	Zhang et al. (2011)
		*in vitro*	Enhanced sensitivity of SKOV3/PTX cells to PTX	Inhibition of β-catenin/c-Myc/Cyclin D1 signaling pathway	Jiang and Hou, (2020)
9	Stomach Cancer	*in vitro*	Increased sensitivity of human gastric cancer BGC-823 and MKN-28 cells to chemotherapeutic agents	Co-optotic effects and down-regulation of chemotherapeutic drug-related genes	Wei et al. (2007)
		*in vitro*	Induction of apoptosis in gastric cancer cells	Autophagy and apoptosis involving the Akt/mTOR pathway	Bai et al. (2018b)
		*in vitro*	Reversing multidrug resistance in gastric cancer cells	Down-regulation of ZNF139, MRP-1 and MDR1 expression	Li et al. (2014a)
		*in vivo*+*in vitro*	Inhibition of gastric cancer BGC-823 cell viability and induction of apoptosis, inhibition of tumor growth	Significantly inhibit cell proliferation through mitochondrial dependent apoptosis	Qin et al. (2013)
		*in vitro*	Enhance the anti-tumor effect of paclitaxel	Inhibition of ROS-dependent Akt pathway and activation of apoptosis pathway in turn	Li et al. (2012a)
		*in vitro*	It not only has a synergistic effect on the cytotoxicity of two gastric cancer cell lines, but also can induce apoptosis	Synergistic effect of apoptosis and down-regulation of chemotherapeutic drug-related genes	Wei et al. (2007)
10	Pancreatic cancer	*in vitro*	Induction of toxicity and apoptosis in pancreatic cancer (PANC-1)	Targeted reactive oxygen species-mediated caspase activation pathway	Wu et al. (2019)
		*in vitro*	Induction of apoptosis in gemcitabine-resistant pancreatic cancer cell line PANC-1	Promote apoptosis by inhibiting PI3K/Akt/mTOR signal pathway, promote autophagy by up-regulating AMPK signal pathway and exert the effect of anti-GEM drug-resistant pancreatic cancer cells	Song et al. (2021a)
		*in vivo*+*in vitro*	It can inhibit the proliferation of pancreatic cancer cells and inhibit pancreatic cancer tumors	Indirectly damage the activity of CDK4/6 and prevent the disorder of cell cycle	Singh et al. (2018)
11	Osteosarcoma	*in vitro*	Inhibition of proliferation of human osteosarcoma cells	Upward adjustment of PTEN pathway	Tian et al. (2017)
		*in vivo*+*in vitro*	Inhibition of proliferation, migration and invasion of human osteosarcoma cells 143B and MG63 cells	Regulation of MAPK/Erk, PTEN/Akt, Juk and Wnt signaling pathways	Wang et al. (2021b)
		*in vitro*	Prevention of multidrug resistance in U-2OS osteosarcoma cell lines	Inhibition of NF-κB signaling pathway suppresses Pgp overexpression	Lu et al. (2017)
		*in vitro*	Induction of apoptosis in U-2OS and MG-63 osteosarcoma cell lines	Induces apoptosis and triggers caspase cascade response through intrinsic and extrinsic pathways	Tao et al. (2014)
12	Cervical Cancer	*in vivo*+*in vitro*	Inhibition of cervical tumor growth and migration *in vitro* and *in vivo*	Upregulation of caspase3 activity induces apoptosis in cervical cancer cells, and Tet combined with MMP2 and MMP9 downregulation inhibits migration and invasion of SiHA cells	Zhang et al. (2019)
		*in vivo*+*in vitro*	Reduced proliferation of HeLa cells and Chinese hamster ovary (CHO) cells stably expressing Eag1 and inhibited tumor growth in mice	Inhibition of Eag1 channel	Wang et al. (2019a)
13	Leukemia	*in vitro*	Induction of cell cycle arrest and megakaryocyte differentiation in acute megakaryocytic leukemia through activation of autophagy	Mediated by the activation of Notch1 and Akt and the subsequent accumulation of ROS	Liu et al. (2017b)
		*in vitro*	Enhanced toxic effects of glucocorticoids on erythromycin-resistant human T-lymphoblast leukemia cells MOLT-4/DNR cells	Inhibition of P-glycoprotein enhances glucocorticoid translocation	Xu et al. (2020)
		*in vitro*	and all-trans retinoic acid have a synergistic effect in promoting HL-60 differentiation and maturation in acute promyelocytic leukemia cells	Expression of MUC1	Liu et al. (2020b)
		*in vitro*	Inhibition of cell viability and induction of apoptosis in a glucocorticoid-resistant human leukemia Jurkat T cell line	Induction of apoptosis by cystein cascade regulation, cell cycle arrest, MAPK activation and PI3K/Akt/mTOR signaling modifications	Wxb, (1087)
		*in vivo*+*in vitro*	Induction of autophagy and differentiation in human leukemia cells	ROS accumulation and inhibition of c-MYC protein expression	Wu et al. (2018)
		*in vivo*+*in vitro*	Inhibit leukemic cell proliferation, induce autophagy and promote cell differentiation	Activation of ROS and Notch1 signals	Liu et al. (2015c)
		*in vitro*	Prevention of resistance of adriamycin to leukemia cells K562	Inhibition of mdr1 gene transcription	Shen et al. (2010)
		*in vitro*	Induction of apoptosis in U937 leukemia cells	Activation of caspase and PKC-delta mediates	Jang et al. (2004)
		*in vitro*	Induction of apoptosis in human leukemia U937 cells	Non-calcium-dependent pathways	Lai et al. (1998)
		*in vitro*	Reversal of multidrug resistance in K562/A02 cells	Inhibition of NF-kappaB activation	Chen et al. (2008)
		*in vitro*	Reversal of MDR in acute leukemia mediated by bone marrow microenvironment	Inhibit the expression of P-glycoprotein	Zhou et al. (2022)
14	Glioma	*in vitro*	Inhibition of migration and invasion of human glioblastoma pleomorphic GBM8401 cells *in vitro*	inhibited several key metastases such as p-EGFR, sOS-1, GrB2, RAS, p-κB-p65, NF-κB-p65, Snail, E-cadherin, N-cadherin, NF-EGFR, SOS-1, GRB2, RAS, p-AKT and p-AKT, NF-AKB, MMP2 and MMP9 related proteins, p-JNK1/2 and p-c-jun ↓, inhibited NF-DNAB binding	Jiang et al. (2018)
		*in vitro*	It significantly inhibited the nuclear translocation and expression of β-catenin and induced the apoptosis of glioma stem cell-like cells	Upregulation of Bax, cleavage of PARP and downregulation of Bcl-2	Zhang et al. (2017b)
		*in vitro*	Inhibits the proliferation of glioma cells and has radiosensitizing effects on glioma cells	Reduces the expression of phosphorylated ERK and its downstream proteins and inhibits the cell cycle in G0/G1 phase	Ma et al. (2017)
		*in vivo*+*in vitro*	Inhibits the growth of human glioma cells and impairs tumor angiogenesis	Reducing the expression of phosphorylated STAT3 and its downstream proteins	Ma et al. (2015)
		*in vitro*	Cytotoxic effect on RT-2 glioma cells, antitumor effect on subcutaneous and intracerebral gliomas, and inhibition of subcutaneous glioma angiogenesis	Inhibit the expression of CD31 and VEGF	Chen et al. (2009a)
		*in vitro*	The combination of caffeine and RT-2 glioma cells significantly reduces the survival rate of RT-2 glioma cells	Increased eIF-2α phosphorylation, decreased expression of cyclin D1, and increased caspase-dependent and non-caspase-independent apoptotic pathways	Chen et al. (2014a)
		*in vitro*	Inhibit the proliferation and invasion of glioma U87 cells	Inhibition of ADAM17 and downregulation of EGFR-phosphatidylinositol-3-kinase (PI3K)-AKT signaling pathway	Wu et al. (2014)
		*in vivo*+*in vitro*	Inhibition of RT-2 glioma growth and angiogenesis in rats		Chen et al. (2009a)
		*in vivo*+*in vitro*	It decreased the total cell viability and induced apoptosis of GBM8401/human glioblastoma cells, and inhibited the growth of subcutaneous tumor in nude mice	Reduced the levels of c-FLIP, MCL-1, and XIAP but increased the signals of cleaved-caspase-3, -8, and -9	Liao et al. (2021)
15	Oral Cancer	*in vitro*	Induction of programmed cell death in human oral cancer CAL 27 cells *via* reactive oxygen species production and caspase-dependent pathways	Associated with beclin-1-induced cellular autophagy	Lien et al. (2017)
		*in vitro*	Induction of apoptosis and autophagy in human oral cancer HSC-3 cells	Induction of apoptosis *via* caspase-8, -9 and -3 and poly (ADP ribose) polymerase dependent pathways and induction of autophagy in human oral cancer HSC-3 cells *via* the beclin-1/LC3-I, II signaling pathway	Yu et al. (2016)
		*in vitro*	Induced death of SAS human oral cancer cells	Activation-dependent apoptosis by cystein and activation-dependent autophagy by LC3-I and LC3-II	Huang et al. (2013)
16	Neuroblastoma	*in vitro*	Induction of apoptosis in human neuroblastoma cells	By regulating the Hippo/YAP signaling pathway	Zhao et al. (2019)
		*in vitro*	Effect of increased radiotherapy on human SH-SY5Y neuroblastoma cells	Associated with partial elimination of radiation-induced G (2)/M accumulation	Chen et al. (2009b)
		*in vitro*	Induction of proliferation and apoptosis in Neuro 2a mouse neuroblastoma cells	Induction of cell cycle arrest and apoptosis through oxidative stress	Jin et al. (2002)
17	Laryngeal Cancer	*in vitro*	Exerts anti-multidrug resistance in Hep-2/v cells	Inhibition of MDR1 overexpression-mediated drug efflux and alteration of hTRA1 and RGS10 expression	Li et al. (2020d)
		*in vitro*	Inhibits the survival and proliferation of CD133 Hep-2 cells	Reduces the number of cells in the S-phase of the cell cycle and promotes apoptosis	Cui et al. (2019)
		*in vitro*	Inhibits the growth of Hep-2 cells	Inhibition of calcium levels and upregulation of Brg1 and AHNAK expression in Hep-2 cells	Cui et al. (2015)
18	Colorectal Cancer	*in vitro*	Inhibition of IL-6-stimulated epithelial-mesenchymal transition in HCT116 cells	Significant downregulation of MMP-2 expression and enzymatic activity in IL-6-stimulated HCT116 cells and restoration of E-calciferol gene promoter activity	Tsai et al. (2021)
		*in vivo*+*in vitro*	Induction of apoptosis and inhibition of xenograft tumor growth in colon cancer cells	Inhibition of Wnt/β-catenin signaling	He et al. (2011a)
		*in vivo*+*in vitro*	Combined ionizing radiation (IR) has a synergistic effect on CT26 in mouse colon adenocarcinoma cells		Lin et al. (2018)
		*in vitro*	Inhibition of epidermal growth factor-induced HT29 cell invasion and migration	EGFR signaling and its downstream molecules	Horng et al. (2016)
		*in vivo*+*in vitro*	Inhibition of lung metastasis in CT26 colorectal adenocarcinoma BALB/c mice		Chang et al. (2004)
19	Endometrial cancer	*in vitro*	Significantly inhibited the proliferation and induced apoptosis of Ishikawa and HEC-1-B cells	Regulation of PI3K/Akt signaling pathway	Shang et al. (2021)
20	Pituitary adenoma	*in vivo*+*in vitro*	*In vitro* inhibits the growth of pituitary adenoma cells and *in vivo* inhibits tumor progression	Induction of autophagy and apoptosis through MAPK/STAT3 signaling pathway	Lyu et al. (2021)
21	Esophageal squamous carcinoma	*in vitro*	Increased sensitivity of human esophageal squamous carcinoma drug-resistant cell line YES-2/DDP to cisplatin	Inhibition of multidrug resistance-associated protein 1	Wang et al. (2012)
22	Renal cell carcinoma	*in vitro*	Inhibition of migration and invasion of human renal cell carcinoma	Regulation of Akt/NF-κB/MMP9 signaling pathway	Chen et al. (2017b)
		*in vitro*	Triggered apoptosis and cell cycle arrest in RCC 786-O, 769-P and ACHN cells	caspase cascade activation and upregulation of p21 and p27	Chen et al. (2014b)
23	Gallbladder Cancer	*in vitro*	Induction of apoptosis in human gallbladder cancer cell line SGC-996 cells	Regulation of Bcl-2/Bax ratio and activation of cleaved cystathione-3 expression	Zhu et al. (2014)
24	Liposarcoma	*in vitro*	Inhibition of proliferation and induction of apoptosis in human malignant liposarcoma SW872 cells	Activation of Caspase-9, downregulation of XIAP and STAT-3 and endoplasmic reticulum stress	Samsuzzaman and JANG, (2022)

### 2.2 Anti-tumor mechanism of action of Tetrandrine

#### 2.2.1 Wnt/β-catenin signaling pathway

Wnt is a protein that activates the Wnt/β-catenin signaling pathway, and the name originates from wingless (a Drosophila somatic node polarity gene) and int (a mouse proto-oncogene), a hybrid of these two homologous genes. The human genome encodes at least 19 Wnt proteins, which activate the Wnt signaling pathway, causing the transcription factor complex to disassemble and release the transcription factor β-catenin, which enters the nucleus and activates gene expression. Accumulation of β-catenin in the nucleus is associated with cancer development (Vallée et al., 2021). Tet inhibits Wnt/β-catenin pathway activity and reduces metastatic tumor antigen 1 (MTA1) expression, preventing metastasis in human hepatocellular carcinoma (HCC) (Zhang et al., 2018). Tet can also inactivate Wnt/β-catenin signaling by downregulating IGFBP-5 expression, inhibit LoVo cell proliferation and induce apoptosis, and suppress tumor growth (Wu et al., 2015a). Tet significantly enhances cisplatin-induced growth inhibition and apoptosis in ovarian cancer cells, causing a redistribution of the cell cycle, which is associated with its regulation of the Wnt/cadherin signaling pathway (Zhang et al., 2011). And it was shown that Tet enhanced the sensitivity of ovarian cancer paclitaxel-resistant SKOV3/PTX cells to paclitaxel (PTX) (Jiang and Hou, 2020), induced apoptosis and inhibited the growth of xenograft tumors in colon cancer cells (He et al., 2011a), all associated with Wnt/β-catenin signaling blockade.

#### 2.2.2 Mitogen-activated protein kinase signaling pathway

MAPK, mitogen-activated protein kinase, or MAP kinase, is a class of serine/threonine kinases. MAPK signaling pathway is a tyrosine kinase receptor-mediated signaling pathway that is widely present in cells from yeast to mammals. The classical MAPK (ERK) involves a cascade reaction of four proteins, Ras-Raf-MEK-ERK, and many tumor cells show abnormal cascade protein transduction, which is an important cause of excessive cell proliferation and tumorigenesis (Dillon et al., 2021). On the one hand, Tet inhibits MAPK signaling pathway, which can block the migration and invasion of nasopharyngeal carcinoma cells NPC-TW-039 (Wu et al., 2020). In addition, it significantly enhances the growth inhibition of nasopharyngeal carcinoma cell lines CNE1, CNE2, and C666-1 by radiation, enhances the radiosensitivity of transplanted tumors, and increases the apoptosis rate of transplanted tumor cells (Wang et al., 2020a). On the other hand, Tet activates the MAPK signaling pathway, inhibits cell viability and induces apoptosis in glucocorticoid-resistant human leukemia Jurkat T-cell lines (Xu et al., 2019). Tet induction of apoptosis and inhibition of colon cancer tumor growth have also been shown to be at least partially associated with activation of the p38MAPK signaling pathway.

#### 2.2.3 Reactive oxygen species signaling pathway

Reactive oxygen species (Ros) in mammalian cells are mainly derived from hydrogen peroxide produced during the folding of mitochondrial electron transport chain, nitrogen oxidase and endoplasmic reticulum proteins (Sarmiento-Salinas et al., 2021). Pathological conditions such as cancer, inflammatory diseases and neurodegenerative diseases have been shown to be associated with excessive production of Ros (Liu et al., 2020a). Lin et al. (2016) reported that Tet caused a g0/g1 phase block in human nasopharyngeal carcinoma npc-tw-076 cells, which increased Ros and ca^2+^ production and eventually led to apoptosis. Tet in human hepatocellular carcinoma induced apoptosis by activating Ros and inhibiting Akt activity to induce apoptosis (Liu et al., 2011), and in human leukemia cells inhibits proliferation and induces autophagy and promotes cell differentiation also due to the accumulation of Ros (Liu et al., 2015; Wu et al., 2018). Tet induces apoptosis in cancer cells by activating the cystatinase pathway, a downstream event of its induction of Ros production, and has therapeutic effects in breast and pancreatic cancers (Wu et al., 2019).

#### 2.2.4 PI3K/Akt/mTOR signaling pathway and NF-κB signaling pathway

PI3K protein consists of a catalytic subunit and a regulatory subunit, which, when bound to tyrosine kinase receptors or cytokine receptors, deregulate the PH structural domain, transactivate Akt proteins, and activate or inhibit the activity of a series of downstream substrates such as apoptosis-associated proteins Bad and Caspase9 by phosphorylation. PI3K/Akt downstream targets are mammalian target of rapamycin (mTOR) proteins. The PI3K/Akt/mTOR signaling pathway is one of the most important intracellular signaling pathways that regulate cell growth, motility, survival, metabolism, and angiogenesis (Yang et al., 2019). PI3K/Akt/mTOR signaling in tumor cells mediates chemoresistance in the tumor microenvironment by shielding immune responses and activating multiple survival signaling pathways in human cancers (Kaboli et al., 2021). Tet downregulates this signaling pathway and inhibits proliferation and invasion of glioma U87 cells (Wu et al., 2014), and also promotes PI3K/Akt/mTOR signaling pathway-mediated cell apoptosis and exert anti-gemcitabine-resistant pancreatic cancer effects (Song et al., 2021a). Bai et al. (2018a) found that Tet induced autophagy and apoptosis in human gastric cancer cells by inducing autophagy and apoptosis involving the Akt/mTOR pathway. Tet inhibited the proliferation of MDA-MB-231 cells and induced autophagy by inhibiting the PI3K/AKT/mTOR pathway (Guo and Pei, 2019).

In addition, Akt in the PI3K/Akt/mTOR signaling pathway can also activate IKK, which has a cross-talk with NF-κB signaling pathway. Core components of NF-κB signaling pathway include nuclear factor κB, NF-κB inhibitory protein, IκB kinase, etc. The signals that activate the NF-κB signaling pathway include the action of signaling molecules such as cytokines (e.g., tumor necrosis factor *α*, interleukins), growth factors (e.g., EGF, PDGF, and NGF), free radicals (reactive oxygen species), and physical signal stimuli such as radiation, as well as pathogenic infections such as bacteria and viruses. During the immune process of the body, NF-κB coordinates many signals that promote cell activation and proliferation, but also those that may lead to inflammation and autophagy in tumorigenesis (Silke and O’REILLY, 2021). Tet ultimately prevents multidrug resistance in U-2OS osteosarcoma cell lines by inhibiting the NF-κB signaling pathway and thereby suppressing P-gp overexpression (Lu et al., 2017). Tet reduces the cell number of SW620 cells and inhibited cell adhesion and migration due to inhibition of nuclear factor-κB, matrix metalloproteinase-2 and matrix metalloproteinase-9 signaling pathways (Juan et al., 2018a). Studies have shown that Tet inhibition of breast cancer angiogenesis and metastasis, as well as reversal of multidrug resistance in the leukemia-resistant cell line K562/A02, are associated with reduced NF-κB activity (Chen et al., 2008; Gao et al., 2013a).

### 2.3 Anti-inflammatory efficacy and mechanism of Tetrandrine

**FIGURE 2:**
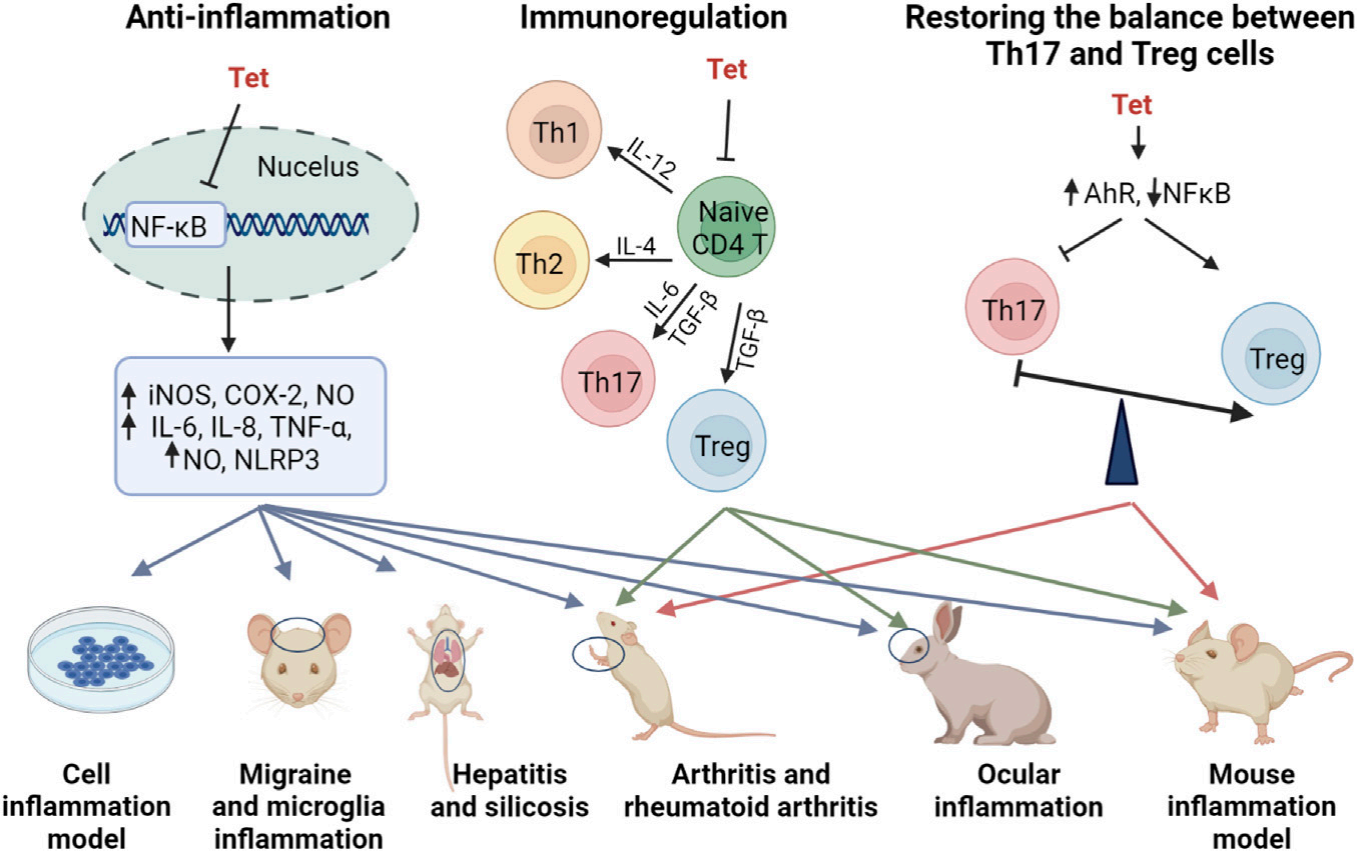
Anti-inflammatory molecular mechanism of Tet.

Tet has some anti-inflammatory effects and exerts good anti-inflammatory effects in a variety of inflammatory models *in vitro* and *in vivo* (Choi et al., 2000a; Feng et al., 2008; Wu et al., 2011; Wu et al., 2015b; Xu et al., 2016a; Yuan et al., 2016a; Feifei and Ming, 2020). In a glial cell inflammation model, Tet inhibited amyloid-β-induced inflammatory cytokines by inhibiting the NF-κB pathway in mouse BV2 microglia (He et al., 2011b). Qin et al. (2018) established a rat model of migraine and found that Tet pretreatment inhibited S100B and p-ERK activation in satellite glial cells of the trigeminal ganglion and attenuated injury perception in rats. In a model of joint inflammation, Tet reduced foot swelling, bone erosion, immunosuppression, and reduced inflammation in a rat model of arthritis and rheumatoid arthritis (Gao et al., 2016a; Jia et al., 2018; Li et al., 2018). The anti-inflammatory effects and anti-inflammatory molecular mechanisms of Tet are comprehensively summarized in Figure 2 and Table 2.

**TABLE 2:** Tet anti-inflammatory effect and mechanism of action.

	Type of inflammation	*In vivo*/*in vitro*	Effectiveness	Mechanism of action	References
1	Migraine rat model	*in vivo*	Reducing injury perception in a migraine rat model	Inhibition of S100B and p-ERK activation in satellite glial cells of the trigeminal ganglion	Guangcheng and Gui, (2018)
2	Hepatitis mouse model	*in vivo*	Protection of mice from hepatitis induced by concomitant knife-bean globulin A	Inhibition of NF-κ b activation to suppress the production of various inflammatory mediators in the liver	Feng et al. (2008)
3	Rat model of severe acute pancreatitis	*in vivo*	Reduce systemic inflammatory response syndrome (SIRS) and multiple organ dysfunction syndrome (MODS) to prevent damage	mediated through the NF-κ b pathway to improve the pro-inflammatory/anti-inflammatory imbalance	Wu et al. (2015c)
4	A model of β-glucan-induced inflammation in macrophages	*in vitro*	Reduction of β-glucan-mediated inflammatory response in macrophages	Inhibition of nf-κ b, ERK and STAT3 signaling pathways	Xu (2016)
5	Mesangial cell inflammation model in rats with glomerulonephritis	*in vitro*	Inhibition of tethered cell activation	Down-regulated ERK/NF- κ b signal transduction and inhibited the expression of inflammatory mediators NO and MMP-9	Chen, (2011)
6	Arthritic mouse model	*in vivo*	Significantly reduced the severity of arthritis and decreased serum levels of pro-inflammatory cytokines	Restoring the balance between Th17 and Treg cells through aryl hydrocarbon receptors	Yuan et al. (2016)
7	Mouse model of ear skin inflammation	*in vivo*	Anti-inflammatory effect	Inhibition of mouse interleukin 5 (mIL-5) and human interleukin 6 (hIL-6)	Choi et al. (2000b)
		*in vivo*	Significantly reduced the level of TNF- *α* in inflamed ears	Increase MSC PGE2 secretion through NF- κ b/COX-2 signal pathway	Yang et al. (2016)
8	Arthritis rat model	*in vivo*	Reduce foot swelling, synovitis and secretion of proinflammatory cytokines in rats	Inhibition of phosphorylation of I κ b α and NF- κ b p65	Gao et al. (2016b)
		*in vivo*	Reduction of bone erosion in rats with collagen-induced arthritis	Inhibition of osteoclast formation by spleen tyrosine kinase	Jia et al. (2018)
9	LPS-induced inflammatory pattern in 264.7 cells pattern	*in vitro*	Blocking nuclear translocation of nuclear factor (NF)-κ b p65 in cells to inhibit IL-6, IL-1β and TNF-*α* expression	Inhibition of i κ b *α* and NF-κ b p65 phosphorylation	Gao et al. (2016a)
10	LPS-induced cartilage-derived ATDC5 cell inflammation model	*in vitro*	Increased secretion of pro-inflammatory mediators and tissue degradation response	Inhibition of i κ b *α* and NF-κ b p65 phosphorylation	Gao et al. (2016a)
11	Mouse inflammation model	*in vivo*+*in vitro*	Anti-inflammatory effect	Inhibits the ability of Th1, Th2, and Th17 cells to differentiate while suppressing the production of Tregs	Zou et al. (2019)
12	Rheumatoid arthritis rat model	*in vivo*	Reduced severity of hindfoot toe swelling in rats, anti-inflammatory, immunosuppressive	Reduced COX-2 expression in rat peripheral blood mononuclear cells and reduced serum concentrations of inflammatory factors	Li et al. (2018)
13	Silicosis mouse model	*in vivo*	Anti-silicosis-associated inflammation	Inhibition of typical and atypical NLRP3 inflammatory vesicle pathways in lung macrophages	Song et al. (2021c)
14	Spinal cord astrocyte injury in rats	*in vitro*	Resistance to injury	Antioxidant and anti-inflammatory activity *via* PI3K/AKT/NF-κB signaling pathway	Bao et al. (2016)
15	Transgenic mouse model of Alzheimer’s disease	*in vivo*	Improving Alzheimer’s disease	Inhibition of microglia inflammatory activation and neurotoxicity in 5XFAD mice	Ren et al. (2021)
16	Rat model of cognitive impairment	*in vivo*	Improve cognitive impairment	Inhibition of inflammation and apoptosis in rats	Ma et al. (2016)
17		*in vitro*	Inhibition of amyloid-β-induced inflammatory cytokines in mouse BV2 microglia	Inhibition of NF- κ b pathway	He et al. (2011)
18		*in vitro*	Inhibited LPS-induced NO release and PGE2 production and attenuated LPS-induced transcription of pro-inflammatory cytokines (TNF-α, IL-4 and IL-8)	Inhibition of COX-2 and iNOS	Wu and NG, (2007)
19	Chronic inflammation model in mice	*in vivo*	Reduced carmine content, granuloma weight, inflammatory cell count and pocket fluid weight in an inflammatory model and inhibited angiogenesis of vascular endothelial cell tube formation	Inhibition of the post-receptor pathway of IL-1alpha and pdgf-bb in chronic inflammation	Kobayashi et al. (1998)
20	Uveitis in rats	*in vivo*	It has obvious inhibitory effect on uveitis induced by endotoxin and interleukin-1 *α* (IL-1 *α*) in rats	Involves multiple inflammatory process pathways and multiple inflammatory mediators	Xiao and Chiou, (1996)
21	Uveitis in rabbits	*in vivo*	Inhibition of uveitis induced by bovine serum albumin in rabbits	Related to the inhibition of cellular and humoral immune function	Xiao et al. (1994)
22	Rat subcutaneous pneumatocystitis model	*in vivo*	Inhibition of vascular permeability, outward migration of neutrophils, beta-glucuronidase (β-G) release and increased superoxide anion (O^2-^) production	Increase SOD activity and cAMP level in neutrophils	He et al. (1989)
23	Rabbit ocular inflammation model	*in vivo*	Anti-inflammatory effect	Inhibition of PGES synthesis	Xiao et al. (1992)
24	Rat model of subcutaneous balloon inflammation	*in vivo*	Inhibition of leukocyte infiltration into airbag, inhibition of monocyte and neutrophil infiltration		Wong et al. (1992)
25		*in vitro*	Significantly inhibited RA-FLS proliferation and triggered apoptosis	Regulate the NEAT1/miR-17-5p/STAT3 pathway and downregulate NEAT1 expression	Duan et al. (2022)

Although inflammation involves the regulation of multiple signaling pathways, MAPK signaling pathway and NF-κB signaling pathway are considered to be the main inflammatory signaling pathways (COGGINS and ROSENZWEIG, 2012). In contrast, Tet mainly regulates inflammation-related pathways such as NF-κB signaling pathway and NLRP3 inflammatory vesicles. Feng et al. (2008) induced hepatitis in mice by injecting them with concomitant cutaneous globulin A (ConA), and treatment with Tet revealed that Tet inhibited the production of various inflammatory mediators in the liver, and the molecular mechanism of action was inhibition of NF-κB activation. Tet reduced hepatitis in a severe acute pancreatitis rat models of systemic inflammatory response syndrome (SIRS) and multiple organ dysfunction syndrome (MODS), again mediated through the NF-κB pathway, improving the pro/anti-inflammatory imbalance (Wu et al., 2015b). As the only drug approved for the treatment of silicosis in China, Tet was found to alleviate silicosis by inhibiting the typical and atypical NLRP3 inflammatory vesicle pathway in lung macrophages (Song et al., 2021).

### 2.4 Clinical application of Tetrandrine

Tet has obvious antitumor, anti-inflammatory and anti-fibrosis effects in cell and animal experiments. Meanwhile, it showed the above functions in clinical trial. In a clinical study, Tet has been proven as an effective medicine for silicosis without other side effects (Miao et al., 2012). It was also demonstrated in another clinical trial of acetylcysteine combined with Tet tablets for silicosis. In addition, the incidence of adverse reactions was significantly lower in patients who took the acetylcysteine Tet tablets, and their lung function was significantly higher after treatment, compared with those in the conventional treatment group (Guo et al., 2020a). In December 2019, COVID-19 virus outbreak made the world trying different methods to fight this pandemic. In China, it was found that traditional Chinese medicine had an obvious effect on COVID-19 virus. Tet as traditional Chinese medicines, could improve the prognosis of COVID-19 patients and reduce the incidence of pulmonary fibrosis in patients during recovery.

The treatment of tumor mainly includes surgical excision and drug chemotherapy. However, most drug chemotherapy has high toxicity and multidrug resistance (MDR), which leads to low survival rate and poor prognosis. One of the complex mechanisms involved in the development of multidrug resistance is the MDR gene and p-glycoprotein, which is also one of the main reasons for the failure of Acute Myelogenous Leukemia (AML) (Cao et al., 2018). Tet is an effective inhibitor of MDR-1 efflux pump. It can reverse MDR in cancer cells by increasing the intracellular concentration of chemotherapeutics drugs (Sun and WINK, 2014). In a clinical trial, Tet combined with daunorubicin, etoposide and cytarabine treated 38 patients with AML. Among them, 36 patients had symptom of hypoplastic bone marrow after chemotherapy, 16 patients (42%) was completely remission or slow recovery, 9 patients achieved partial response (PR), and 13 patients treatment failure (Xu et al., 2006).

In addition, Tet has antihepatic fibrosis (Teng et al., 2015; Zhang, 2016) and cardiomyocyte fibrosis (Gan et al., 2018) and has been used to treat silicosis with fibrosis. In addition, Tet (Yu et al., 2001; Meng et al., 2008; Pinelli et al., 2010; Xu et al., 2010; Huang et al., 2011; Zhang et al., 2017a; Li et al., 2020) protects the cardiovascular system through mechanisms such as antihypertensive, antiarrhythmic and anti-myocardial ischemia and reperfusion injury. Also, Tet has antibacterial (Lee et al., 2012; Li et al., 2020) and activates mesenchymal stem cells to enhance immunomodulation (Yang et al., 2016).

## 3 Chemical-based structural modification of Tet

**FIGURE 3:**
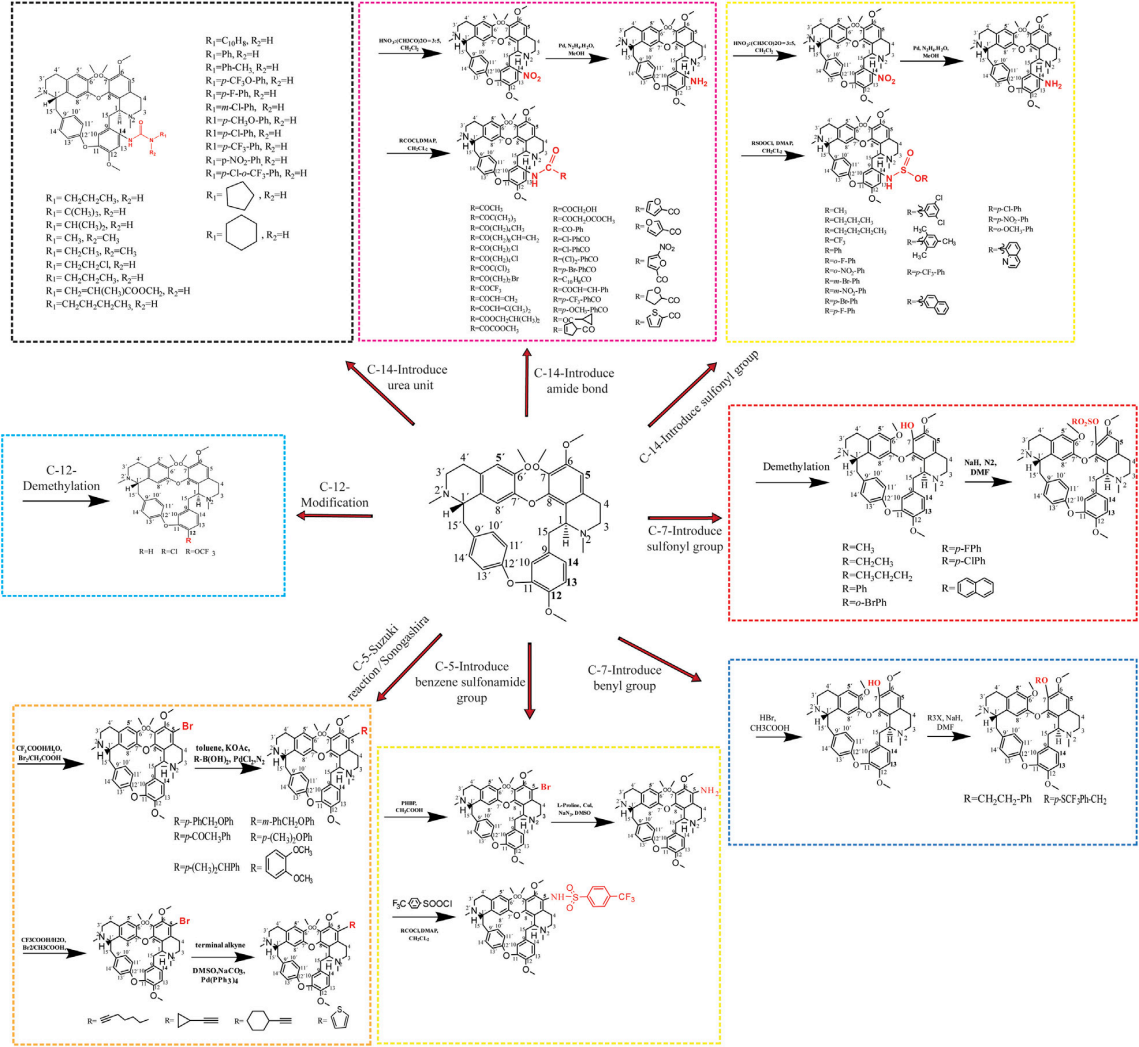
Modification of the chemical structure of Tet.

The introduction of key chemical groups to the original structure of a compound can modulate its key pharmacological properties, such as metabolism or efficacy, among others, without the need to develop or redesign the synthetic method as well as restart the synthesis (Moir et al., 2019). Tet (6,6′,7,12-tetramethoxy-2,2′-dimethyl-(1β)-berbaman), whose structure belongs to the structure of Tet (6,6′,7,12-tetramethoxy-2,2′-dimethyl-(1β)-berbaman) is a bis(benzylisoquinoline) alkaloid with molecular formula C_38_H_42_N_2_O_6_, molecular weight 622.76 g/mol and CAS No. 518-34-3. The structural formula of Tet in the figure below shows that the multiple methoxyl groups on the benzene ring make the benzene ring electron-rich and its electron cloud density is in the order of C-5 position > C-14 position > C-5′ position > C -12 position. Secondly, the tertiary nitrogen atoms on the isoquinoline ring are more basic and can easily form salts in the presence of acid, and can also form quaternary ammonium salts in the form of haloalkanes, so the derivatization reactions of Tet, i.e., structural modifications, mostly occur at the above-mentioned sites (Xiangyu et al., 2022). The chemical structure modification of Tet is summarized in Figure 3.

### 3.1 Structural modification of the C-5 position

The C-5 position of hanbanzide is mostly reacted with halogen substitution as the first step, and then other key pharmacophore groups are introduced, usually aryl groups, terminal alkynyl groups, heterocycles containing nitrogen and sulfur atoms, etc. Similar to the Sonogashira cross-coupling reaction, the Suzuki reaction is a cross-coupling reaction of aryl halides with aryl boronic acids and is a widely known and versatile method used to construct C-C bonds. Niu et al. (2019) synthesized 12 5-alkynyl-Tet derivatives by the Sonogashira cross-coupling reaction and found that most of the derivatives had better anti-tumor activity than Tet. The mechanism of action was related to the triggering of an intrinsically activated apoptotic pathway, upregulating pro-apoptotic genes (Bax, caspase-3), downregulating anti-apoptotic Bcl-2 and releasing Cyt-C. Wei et al. (2016) synthesized 15 new Tet by the Suzuki reaction using 5-bromohampanoid alkaloids as key intermediates. The *in vitro* evaluation of anti-HL60 and A549 cellular activity showed that the anticancer activity was enhanced by the introduction of heteroatomic thiophene derivatives and acetylated acetyl phenyl derivatives. (Wu et al., 2013) also synthesized 20 new powdered hamphenine derivatives by combining the Sonogashira and Suzuki reactions using 5-bromohamphenine as the key intermediate. The introduction of an aromatic ring group at the C-5 position significantly increased the proliferative activity of Tet against human breast cancer cell line (MCF-7) and hepatocellular carcinoma cell line (HepG2), compared with sunitinib up to 29.2-fold, with a mechanism of action related to the induction of apoptosis in cancer cells (Li et al., 2017). In addition, the introduction of sulfonamide group at the C-5 position was effective in inducing multiple myeloma cell (RPMI-8226) death, and in comparison to hanpacryl methacin itself, the 5-(4-trifluoromethylbenzenesulfonamido)-hanpacryl methacin derivative had significant anti cell line activity, with nearly 2-fold increased activity against myeloma RPMI-8226 cell line (Wang et al., 2022).

### 3.2 Structural modification of C-14 position

The nitration reaction of C-14 position of Hanbanzin, catalyzed by Pd/C, is reduced to amino group with hydrazine hydrate (N2H4-H2O), and acylation or sulfonylation with a series of acyl halides and carboxylic acid compounds or sulfonyl chlorides to obtain Tet derivatives of amide or sulfonamide. The introduction of the urea unit on C14 of Tet enhances the antitumor activity of Tet, with derivatives having up to 12-fold the anticancer activity of Tet, 31-fold that of 5-fluorouracil, and 26-fold that of cisplatin, with a mechanism of action related to the induction of cell death by the endogenous apoptotic pathway (Lan et al., 2018). The introduction of an amide bond on C14 of Tet was shown to enhance the cytotoxic effect of Tet on hepatocellular carcinoma cell lines MHCC97L and PLC/PRF/5, the most potent derivative of antitumor activity, 15.8-fold that of Tet and 30.3-fold that of sorafenib, with a mechanism of action endoplasmic reticulum stress-related apoptotic pathway and activation of JNK and caspase pathways associated (Lan et al., 2017), where the introduction of electron-absorbing groups (-F, -CI, -Br) at the C-14 position has higher antitumor activity than electron-donating groups (-OH, -OMe), and amide side chains with aromatic ring substitutions have superior antitumor activity than mono- or multi-substituted benzene ring derivatives. The introduction of sulfonamide groups at the C-14 position significantly enhances the anticancer activity of sulfonamide-antihexine derivatives compared to the parent compounds. Among them, the derivatives in which the benzene ring was substituted with an electron-absorbing group at the 14-amino position showed a stronger increase in antiproliferative activity, and the mechanism of action may be related to the increased expression of the apoptotic protein Bax and a corresponding decrease in the expression of the anti-apoptosis-related proteins (Bcl-xl and Bcl-2) (Song et al., 2018).

### 3.3 Structural modification of the C-7 position

The introduction of fluorinated benzyl bromide into Tet increased the inhibitory activity against A549 lung cancer cells while reducing the cytotoxicity against the human normal hepatocyte line HL-7702 Gao et al. (2021) while Tet was first demethylated to synthesize the antihexenolines, after a series of reactions, and then different benzyl bromide groups were added to the antihexenoline structure, three of the ten derivatives obtained were comparable to the antihexenoline The other 7 sulfonyl analogs showed stronger antitumor activity than the parent compound, and the most active compound was up to 12.15 times that of Tet.

### 3.4 Structural modification of the C-12 position

The methoxy group at the para position of C-12 is the key group that induces the hepatotoxicity of Tet. Reducing CYP3A4-mediated toxicity by replacing or eliminating the metabolically unstable C12-methoxy group is one of the important means to improve the clinical application of Tet and explore Tet derivatives with more efficient antitumor activity. Demethylation of the methoxy group at the 12-position of TetC or by a metabolically stable trifluoromethoxy or chloro substituent directly inhibits the proliferation of vincristine-resistant leukemia cells (VCR-RCEM), and its mechanism of action may disrupt mitochondrial membranes potential, affecting early apoptosis-related (Schütz et al., 2020).

### 3.5 Forming salts

The solubility of a drug will affect its pharmacokinetic properties, chemical stability and the choice of dosage form, which is an important content in the evaluation of druggability. Salt formation can change the solubility of the drug, improve the water solubility of the drug, improve the compliance of the drug, and improve its stability through salt formation. After the tetrandrine is formed into a salt, on the one hand, the influence of the lipid partition coefficient of the derivative on the cytotoxicity is improved; on the other hand, the defect of its own low solubility is changed to meet the selection of different dosage forms. The Tet structure contains two tertiary nitrogen atoms with strong basicity, which are easy to form salts with acids or quaternary salts with halogenated alkanes. Using 1M HCl (inmethanol) and CH_2_Cl_2_ as reaction conditions, tetrandrine hydrochloride was synthesized. Compared with tetrandrine itself, it could significantly inhibit the proliferation activity of HEL, K562, MDA-MB-231, PC3, and WM9 cells (Lan et al., 2018). Tetrandrine citrate is synthesized by tetrandrine and citric acid in a ratio of 4:1. The solubility in water reaches 500 mg/ml. It is a new type of oral active tetrandrine salt, which can inhibit ima. The proliferation of tinib (IM)-resistant chronic myeloid leukemia (CML) cells without obvious toxicity in a nude mouse xenograft model may be related to the loss of p210Bcr-Abl and β-catenin proteins (Xu et al., 2012).

## 4 Structural modification of tetrandrine based on nano-carrier

In recent years, with the vigorous development of new technologies and new materials, the development of nanomaterials has attracted wide attention. Nanomaterials are defined as materials with inherent quantum limitations in at least one dimension, which is also reflected in their electronic structure and other physical properties (Alfieri et al., 2022). Compared with ordinary pharmaceutical preparations, nanomaterials have the following advantages as drug carriers: 1) they can improve their pharmacokinetics by changing the physical and chemical properties of drug molecules (water solubility, fat solubility, etc.). Help drug molecules cross physiological and pathological barriers, thus improving bioavailability (Shi et al., 2017); 2) it can achieve active and passive targeting, increase the local concentration of nano-drugs in the focus, improve the efficacy and reduce the occurrence of side effects, so as to achieve a more safe and effective diagnosis and treatment of diseases (Petros and Desimone, 2010); 3) reduce immune recognition and clearance of reticuloendothelial system by ingenious “camouflage”, protect active molecules from enzymolysis, increase drug retention time, prolong drug half-life, and enhance drug efficacy (Lu et al., 2016); 4) prolong drug circulation time, control drug release, and improve patient compliance; 5) simultaneous visualization of tumor therapeutic effects based on new imaging techniques; 6) to achieve diversity and intelligence. In addition to bioactive chemical molecules, nano-carriers can also contain bioactive substances such as peptides and nucleic acids, as well as contrast agents outside clinical treatment; 7) the drug was delivered to cancer cells by endocytosis, so that the cytotoxicity was not decreased due to the decrease of intracellular pH, and the physiological drug resistance induced by pH was reversed (Li et al., 2011b).

**FIGURE 4:**
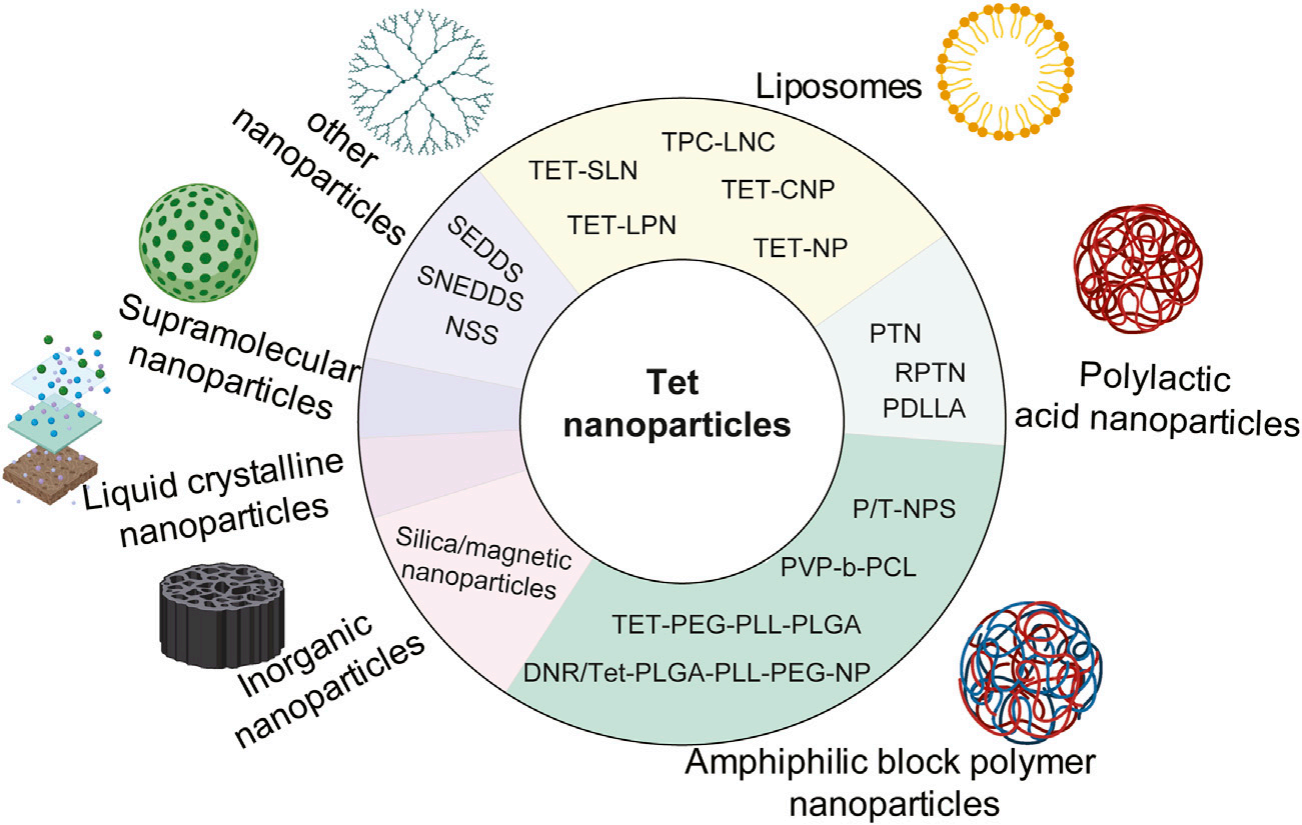
Structural modification of Tet based on nano-carrier.

Nanoparticle-drug delivery system is an indispensable new drug delivery system, including organic nanoparticles (for example, polymer nanoparticles, dendrimer nanoparticles, liposomes, micelles, solid lipid nanoparticles, and hydrogels, etc.), inorganic nanoparticles (metal, magnetic, and semiconductor nanoparticles, and carbon nanotubes, etc.). And some nanocrystals designed to improve the rate of dissolution and absorption (Zhang et al., 2020). At present, in order to improve the water solubility, targeting, bioavailability and stability of Tet, many nanomaterials have been developed and utilized, including liposomes, polymer nanoparticles, supramolecular nanoparticles, inorganic nanoparticles, and liquid crystal nanoparticles. The structural modifications of Tet based on nanosystem delivery are summarized in Figure 4.

### 4.1 Liposomes

Liposomes are nanomaterials formed by hydrophilic cores and hydrophobic lipid bilayers (Van Der Koog et al., 2021). Liposome nanoparticles are multicomponent lipid systems, which usually contain phospholipids, ionizable lipids, cholesterol and polyethylene glycol lipids. The traditional type of liposome nanoparticles refers to liposomes, which was first proposed by British hematologist AlecDBangham in 1961. Importantly, liposomes may overcome the limitations of a single Tet due to the following characteristics: 1) liposomes are amphiphilic and can disperse Tet to avoid aggregation; 2) liposomes are multi-functional nano-carriers that can be targeted and triggered by multiple stimuli because of their surface modification flexibility, size control and adjustable encapsulation. 3) Liposomes avoid drug exposure to normal tissues, organs, and blood circulation, so as to prevent drug degradation, immune reaction and toxicity before reaching the desired lesions; 4) liposomes are composed of natural phospholipids. It is a biocompatible and biodegradable material, minimizing the risk caused by formula. 5) Compared with free Tet, liposome preparation can improve the therapeutic efficiency by prolonging tissue penetration and retention. (Cheng et al., 2021).

Fan et al. (2013) prepared spherical liposomes by pH gradient loading method, which enhanced the local delivery of Tet, thus enhancing the anti-arthritis effect of Tet. Li et al. (2011c) prepared Tet solid lipid nanoparticles (SLNs) by melt emulsification and ultrasound. It was found that TET-SLN had higher concentration in plasma, lower clearance rate, higher release rate and was absorbed by reticuloendothelial system organs. Li et al. (2014b) prepared cationic solid lipid nanoparticles (TET-CNP) and solid lipid nanoparticles (TET-NP) by emulsion evaporation-curing method at low temperature, which were used to load Tet. Compared with Tet ophthalmic solution (TET-SOL), they delayed the release of Tet and significantly improved their bioavailability. Li et al. (2006) used ultrasonic treatment to prepare solid lipid nanoparticles (SLN), which were used to deliver Tet. Stability evaluation found that SLN after ultrasonic treatment was more stable, simple and efficient. Zhao et al. (2013) prepared lipid nanocapsules (TPC-LNCs) loaded with Tet-phospholipid complex by phase inversion method. Compared with traditional tablets, oral bioavailability was greatly improved. Tet was encapsulated with nanoliposomes and g DSPE-MPEG 2000 (DP) was added as a stabilizer, and the results showed that the nanoliposomes could significantly improve the physicochemical properties of Tet, making it safer and more efficient (Song et al., 2022).

As an excellent multi-functional nano-drug carrier, liposomes are widely used to enhance tumor targeting (Perche and Torchilin, 2013). The iRGD peptide modified lipid-polymer hybrid nanosystem (LPN) prepared by Zhang et al. (2017c) is a targeted liposome nano-carrier for the co-delivery of PTX and Tet. This system is more toxic to cells than free drug combinations and non-targeted LPN, significantly induce apoptosis of drug-resistant cells, and has the advantages of high drug loading rate and good stability. Transferrin is a kind of plasma glycoprotein bound to iron. Vincristine plus Tet liposome modified by transferrin is also a kind of targeted liposome, which can significantly prolong the circulation time, make the drug accumulate obviously in the part of brain tumor, and overcome the multidrug resistance of malignant glioma cells (Song et al., 2017).

### 4.2 Polymer nanoparticles

Nano-polymer refers to the polymer with at least one-dimensional size within 100 nm, including spherical, linear, tubular and other structures. Polymer nanoparticles are an important part of nano-polymers. They have stable structure and can be designed and prepared at the molecular level, such as the selection of polymerization methods or monomers. Because their size and particle uniformity can be controlled, they not only have small size effect, surface effect and quantum tunneling effect, but also have other functions such as temperature, pH, electric field and magnetic field (Xiaobei, 2014). Polymer nanoparticles mainly include poly (DL-lactide-glycolide), polyethylene glycol, poly (lactic acid), poly (vinyl alcohol), Polyaniline, polypyrrole, polyethyleneimine and so on.

#### 4.2.1 Polylactic acid nanoparticles

Poly (lacticacid) (PLA) is a polymer structure formed by dehydration and condensation between individual lactic acid molecules. Its molecular formula can be expressed as (C_3_H_4_O_2_) n, and its molecular weight is determined by different degrees of polymerization (Guoqiang and Kai, 2002). FDA (FoodandDrugAdministration) approved polylactic acid and its derivatives as pharmaceutical excipients in 1995. Polylactic acid is widely used in the field of drug release, surgical suture materials, biomedical instruments and so on (Gang and FENG, 2003; Siripitayananon, 2005; Musumeci et al., 2006; Gao et al., 2014). At present, poly (lactic acid) or poly (lactide) (PLA) is the most widely studied and used biodegradable and renewable thermoplastic based polyester, which has great potential to replace traditional petrochemical-based polymers (Bhuvanesh and Revagade, 2007; Rasal et al., 2010; Saeidlou, 2012).

Meng et al. (2016) found that drug release and cell uptake can be accurately controlled simultaneously by adjusting the surface charge of polylactic acid-glycolic acid (PLGA) nanoparticles (TPNs) multilayer coatings containing Tetrandrine. Shi et al. (2015) prepared polylactic acid-glycolic acid copolymer (PLGA) nanoparticles by emulsion solvent diffusion method. At the same drug concentration, the nanoparticles showed better performance than pure drugs. Among them, the inhibitory effect of DMAB stable particles on A549 cells was the strongest, while that of PVA stable particles was the weakest. (Que et al. (2019) found that compared with the above TET-PLGA nanoparticles (PTN), the erythrocyte membrane camouflaged TET polylactic acid-glycolic acid copolymer (RPTN) nanoparticles (RPTN) significantly reduced the swallowing of RAW264.7 macrophages due to the retention of natural membrane proteins. The combination of TET RPTN and adriamycin (ADR) significantly enhanced the sensitivity of drug-resistant cells MCF-7/ADR to ADR. Racemic polylactic acid (PDLLA) membrane is a new type of drug sustained release and anti-adhesion material *in vivo* and *in vitro*, which can significantly inhibit the proliferation and collagen synthesis of fibroblasts, improve the local bioavailability of Tet, and induce apoptosis and death of fibroblasts (Yao et al., 2020).

#### 4.2.2 Amphiphilic block polymer nanoparticles

In drug delivery systems, polymer nanoparticles with amphiphilic diblock or triblock polymers as drug carriers are the mainstream in the field of drug delivery systems. These nanoparticles can escape the clearance of the reticuloendothelial system (RES) and prolong circulation in blood vessels, so they accumulate in the tumor tissue through the EPR effect (Van Vlerken et al., 2007; Zhang et al., 2011c). Compared with free Tet, Tet loaded nanoparticles Tet-NP could more effectively inhibit the proliferation and induce apoptosis of osteosarcoma cells (Tian et al., 2016a). Poly (lactide-co-glycolide) nanoparticles without surfactant were prepared from PLGA by nano-precipitation method, and Tet was encapsulated in it, which can significantly improve the anticancer activity of Tet (Shi et al., 2016b). The nano-drug delivery system based on PEG-PLL-PLGA polymer, carriers daunorubicin (DNR) and Tet, were injected into nude mice carrying MDR leukemia cell K562/A02 xenografts, targeting reversing multidrug resistance MDR (Guo et al., 2015). DNR/Tet-PLGA-PLL-PEG-NP copolymer nano-carriers were constructed by improved double emulsion solvent evaporation/separation technique, and further modified with transferrin (Tf), which improved the solubility of DNR and Tet and increased the targeting of drugs to tumors (Liu et al., 2015d). Xu et al. (2016b) reported that spherical core-shell Tet loaded nanoparticles were prepared by nano-precipitation method using amphoteric poly (N-vinylpyrrolidone)-block-poly (ε-caprolactone) (PVP-b-PCL) copolymers. Tet was incorporated into NP with high encapsulation efficiency and released continuously. Compared with free Tet, the toxic effect of Tet-NP on A549 cells was stronger and positively correlated with dose and time. The ability to induce apoptosis is also stronger.

Li et al. (2010) reported for the first time a Trojan strategy, that is, a simple method to produce nanoparticles containing Tet based on amphiphilic block copolymers, and the results *in vitro* showed that compared with the same dose of free Tet (1–8 μg/ml), Tet-np at lower concentration (1–8 μg/ml) significantly inhibited the proliferation of cancer cells, and the stability, solubility and anti-tumor activity of Tet were improved. It has been found that the co-delivery of Tet and Ptx in mPEG-PCL nanoparticles has a significant anti-hepatoma effect (Li et al., 2012b), however, it is difficult for drug-loaded nanoparticles to penetrate into the tumor mass and inhibit the growth of cancer cells far from the injection site (Emerich et al., 2002; Ding et al., 2011). The mPEG-PCL nanoparticles (P/T-NPs) co-loaded with Tet and PTX were encapsulated into physically cross-linked gelatin hydrogel and then implanted into the tumor site, the drug could be continuously released. The results showed that P/T-NPs inhibited the growth and invasion of BGC-823 gastric cancer cells more effectively than free drugs or non-Rigner combination (Zhang et al., 2016b).

### 4.3 Inorganic nanoparticles

Inorganic nanoparticles mainly include metal nanoparticles (Ag, Au, Zn, Co., MoS, Ni, CuS, Gd, TiO_2_, Fe, etc), magnetic nanoparticles (FeO, Fe_3_O_4_, FeCo, FeSe_3_, etc), semiconductor nanoparticles (Mn, C_3_N_4_-BiOCl, TiO_2_, CuS, etc.) and carbon nanotubes (Amidated carbon nanotube, Carboxylated multi-walled carbon nanotube, Hydroxylated single-walled carbon nanotube, etc) (Zhang et al., 2020). Multifunctional mesoporous silica nanoparticles were prepared by self-assembly *in situ* loading method, and a co-delivery system of antineoplastic drug PTX and multidrug resistance reversal agent Tet was established. This system inhibited the growth of tumor cells more effectively than only delivering PTX or free PTX, and the drug-loaded nanoparticles completely reversed the resistance of MCF-7/ADR cells to PTX at the molar ratio of 4.4 PTX/TET to PTX. The resistance reversal index was 72.3, and the mechanism was related to cell cycle arrest (Jia et al., 2015).

Wang et al. (2019b) proposed a new MACS ®technique for purification of magnetic nanoparticles for the first time. PLGA-based multifunctional nanoparticles were successfully synthesized and purified by co-encapsulation of Tet and magnetic materials (Fe_3_O_4_), which improved the encapsulation efficiency and anti-proliferation effect on A549 lung cancer cells. The mechanism is related to activating the mitochondrial pathway and inducing A549 cell apoptosis by loading lysosomes. Shi et al. (2016c) developed poly (lactic acid-glycolic acid) particles co-loaded with Tet-magnetite, which is a novel polymer magnetic delivery system that releases Tet to inhibit cancer-related TASK3 channels in a dose-dependent manner.

### 4.4 Liquid crystalline nanoparticles

Liquid crystalline nanoparticles (LCNPs) are formed by amphiphilic lipids in the presence of excess water, which provide higher encapsulation than other drug delivery systems, and they have a higher proportion of lipids in the particles, so they have a larger surface area, so they are proposed as potential drug delivery carriers (Avachat and PARPANI, 2015). Liu et al. (2016) found that Tet-LCNPs, as a new ophthalmic drug delivery system for Tet, compared with Tet ophthalmic solution, has many advantages, such as increasing drug solubility, better sustained release effect, significantly promoting corneal penetration of Tet and improving bioavailability.

### 4.5 Supramolecular nanoparticles

PTX-SA-RGD is a new type of supramolecular nanomaterial formed by coupling paclitaxel (PTX) with tumor-specific peptide RGD (arginine-glycine-aspartic acid) and succinic acid (SA). Li et al. (2020e) constructed carrier-free nanofibers using PTX-SA-RGD as drug carriers, and obtained nanofiber drug delivery systems co-loaded with PTX and Tet, which improved the poor solubility of PTX and Tet. The toxic effect of their combination on tumor cells was significantly enhanced, and the mechanism was related to the induction of mitochondrial apoptosis.

### 4.6 Other nano-carrier systems

SNEDDS is an isotropic mixture of oil, surfactant, hydrophilic cosurfactant and drug substances. When introduced into the aqueous medium, fine oil-in-water microemulsion is formed under the gentle agitation of digestive movements of the stomach and intestines. SNEDDS is a relatively new term used to describe preparations whose pellet size is smaller than 100 nm. Self-emulsifying drug delivery system (SEDDS) is a relatively new, lipid-based technological innovation, which has a great prospect in improving the oral bioavailability of drugs (Singh et al., 2009). Liu et al. (2018) improved the dissolution and oral bioavailability of Tet through self-nano-emulsified drug delivery system (SNEDDS), and the oral bioavailability was about 2.33 times higher than that of commercial tablets. In the rapidly developing nano-drug delivery system, nano-suspension (NSS) is a favorable strategy in drug design for cancer treatment, which can overcome the shortcomings of other nano-carriers, such as poor physical stability, low drug loading and low encapsulation ability. Guo et al. (2020b) prepared Tet-NS nano-suspension by wet grinding. The experimental results showed that the cumulative dissolution of Tet-NS increased by 4–5 times within 2 h, which increased the solubility of Tet. High concentration of Tet-NS significantly induced apoptosis and cell uptake of A549 cells.

## 5 Conclusion

Tet, as a natural product from the Chinese herbal medicine Fangji, is an alkaloid with multiple pharmacological activities, and we focus on the anti-tumor and anti-inflammatory activities of Tet and its current clinical applications and the problems encountered at present. Tet is currently used in clinical practice mainly for the treatment of silicosis, hypertension, and cardiac arrhythmias, but modern research has shown that it also has good anti-tumor, anti-inflammatory, antioxidant, anti-bacterial, anti-viral, and immunity-boosting effects. In up to 24 tumor types, Tet has been shown to have a significant effect through Wnt/β-catenin signaling pathway, MAPK signaling pathway, Reactive oxygen species signaling pathway, PI3K/Akt/mTOR signaling pathway and NF-κB signaling pathway mediating its role in tumors. In addition, Tet also has anti-inflammatory effects, and the mechanism by which it exerts its anti-inflammatory effects is through mediating the NF-κB signaling pathway and CD4 T-cells, upregulating the levels of INOD, COX-2, NO, IL-6, IL-8, TNF-a, NLRP3, Th1, Th2, Th17 and Treg factors, and by acting on AhR and NF-κB signaling pathway to restore the balance between Th17 and Treg cells. Thus Tet is a natural product with great potential for clinical application development.

Despite its multiple pharmacological activities, the disadvantages of Tet, such as its low bioavailability and side effects in humans, have limited its clinical application. Therefore, many researchers have designed and synthesized new Tet derivatives by structurally modifying the C-5, C-14, C-7, and C-12 positions to improve their pharmacokinetic and therapeutic properties. In addition to chemical structural modifications, delivery *via* nanosystems is also an important way to improve Tet. In this review, liposomal nanoparticles, polymeric nanoparticles, inorganic nanoparticles, liquid crystalline nanoparticles, novel supramolecular material nanoparticles and other nanoparticles are shown to have potential in improving Tet’s clinical application limitations.

In general, we comprehensively summarized the latest research progress on the pharmacological mechanism of Tet in anti-tumor and anti-inflammation, as well as the current clinical application and problems of Tet. The latest progress in the improvement of its physical and chemical properties (water solubility, fat solubility, etc.) by chemical structural modification and structural modification based on nanosystem delivery was also summarized. We hope that this review will help to discover the new pharmacological activities of Tet, expand its clinical application in tumors and inflammatory diseases, and help researchers modify the structure of other active natural products, so that they can be better used in the treatment of clinical diseases.
